# Glycogene Expression Alterations Associated with Pancreatic Cancer Epithelial-Mesenchymal Transition in Complementary Model Systems

**DOI:** 10.1371/journal.pone.0013002

**Published:** 2010-09-27

**Authors:** Kevin A. Maupin, Arkadeep Sinha, Emily Eugster, Jeremy Miller, Julianna Ross, Vincent Paulino, Venkateshwar G. Keshamouni, Nhan Tran, Michael Berens, Craig Webb, Brian B. Haab

**Affiliations:** 1 Van Andel Research Institute, Grand Rapids, Michigan, United States of America; 2 The Translational Genomics Research Institute, Phoenix, Arizona, United States of America; 3 Department of Internal Medicine, University of Michigan Medical School, Ann Arbor, Michigan, United States of America; Universidade de São Paulo, Brazil

## Abstract

**Background:**

The ability to selectively detect and target cancer cells that have undergone an epithelial-mesenchymal transition (EMT) may lead to improved methods to treat cancers such as pancreatic cancer. The remodeling of cellular glycosylation previously has been associated with cell differentiation and may represent a valuable class of molecular targets for EMT.

**Methodology/Principal Findings:**

As a first step toward investigating the nature of glycosylation alterations in EMT, we characterized the expression of glycan-related genes in three *in-vitro* model systems that each represented a complementary aspect of pancreatic cancer EMT. These models included: 1) TGFβ-induced EMT, which provided a look at the active transition between states; 2) a panel of 22 pancreatic cancer cell lines, which represented terminal differentiation states of either epithelial-like or mesenchymal-like; and 3) actively-migrating and stationary cells, which provided a look at the mechanism of migration. We analyzed expression data from a list of 587 genes involved in glycosylation (biosynthesis, sugar transport, glycan-binding, etc.) or EMT. Glycogenes were altered at a higher prevalence than all other genes in the first two models (p<0.05 and <0.005, respectively) but not in the migration model. Several functional themes were shared between the induced-EMT model and the cell line panel, including alterations to matrix components and proteoglycans, the sulfation of glycosaminoglycans; mannose receptor family members; initiation of O-glycosylation; and certain forms of sialylation. Protein-level changes were confirmed by Western blot for the mannose receptor MRC2 and the O-glycosylation enzyme GALNT3, and cell-surface sulfation changes were confirmed using Alcian Blue staining.

**Conclusions/Significance:**

Alterations to glycogenes are a major component of cancer EMT and are characterized by changes to matrix components, the sulfation of GAGs, mannose receptors, O-glycosylation, and specific sialylated structures. These results provide leads for targeting aggressive and drug resistant forms of pancreatic cancer cells.

## Introduction

Pancreatic cancer has one of the poorest survival rates of any major cancer [1]. The extreme lethality of pancreatic cancer is related to its tendency to disseminate at early stages prior to diagnosis [2], [3] and its resistance to chemotherapeutics [2], [4]. The acquisition of migratory and drug-resistant traits in pancreatic cancer cells may occur in a step-by-step fashion, accompanied by increasing changes to the genetics and morphologies of the cancer cells. Early-stage and pre-malignant states are thought to consist of dysplastic cells within pancreatic ducts [5], and the progression to ductal adenocarcinoma is characterized by proliferating epithelial cancer cells assembled in tube-like ductal structures surrounded by fibrotic stroma. The metastatic dissemination of pancreatic cancer requires cells to break away from the epithelial ductal structures and take on characteristics of migratory, mesenchymal cells. This transition involves enormous remodeling of the cell and is likely driven by genetic aberrations, extracellular signals, and the activation of differentiation programs in the cancer cells. Characterizing the molecular alterations associated with the phenotypic switch in pancreatic cancer cells from epithelial-like to mesenchymal-like traits will provide insights into avenues for detecting and targeting this conversion.

This major phenotypic switch in pancreatic cancer cells may be driven by the epithelial-mesenchymal transition (EMT). EMT is a biological program that coordinates the conversion of cell differentiation from the epithelial characteristics of strong cell-cell adhesion, polarity, and smooth morphology to the mesenchymal characteristics of minimal cell-cell contacts, loss of polarity, and increased cell projections [2], [6], [7], [8], [9]. The EMT is normally activated in development and wound healing during tissue remodeling, but it is thought to be abnormally activated by certain types of cancer cells to confer the traits associated with highly lethal cancers. Multiple lines of evidence support the importance of EMT in promoting pancreatic cancer aggressiveness. The general histological loss of cellular differentiation is a highly accurate predictor of poor outcome in pancreatic cancer [10], [11], and the specific EMT markers of reduced E-cadherin and increased vimentin correlate with poor survival [12], [13] and invasion [14]. Mouse models of pancreatic cancer recapitulate that relationship [15]. In agreement with those findings, the induction of a transcription factor called snail, which controls E-cadherin repression, results in increased metastasis and chemoresistance of pancreatic cancer cells [16]. Furthermore, the mesenchymal-like cancer cells may be more drug-resistant than their epithelial-like counterparts, as suggested by the correlation between Gemcitabine-resistance and mesenchymal traits [17] and the loss of EGFR-inhibitor sensitivity in pancreatic cancer cells that have lost epithelial-like traits [8]. Intensive investigations have uncovered many of the regulatory mechanisms and molecular characteristics of this conversion [2], [6], [7], [8], [9], but the in-vivo factors at work in cancer EMT and that are relevant to pancreatic cancer progression are not clear.

The glycosylation of a cancer cell may be significantly remodeled during EMT, although the nature of this association has not been well characterized. Glycosylation is a dynamic process involving a concerted interplay between various glycosyltransferases and associated enzymes in the endoplasmic reticulum and Golgi apparatus [18]. Glycan structures are involved in proper protein folding [19], [20], [21], intracellular trafficking [18], [20], [22], cell growth and differentiation, [20], [23], adhesion and migration [18], [24], and cell-based immunity [20], [25], among other functions. Changes in glycans have been detected and implicated in various pathologic conditions [23], [26]. In addition, the carbohydrate structures on cell-surface and secreted proteins are good indicators of cell type and status, as they specifically change in association with development [27], [28], cell differentiation and activation [29], [30], and transformation [31]. Therefore we hypothesized that pancreatic cancer EMT is characterized by specific glycosylation alterations that play functional roles in cancer cell differentiation or migration.

As an initial step in testing this hypothesis, we examined the expression of glycosylation-related genes in three model systems of EMT or migration. Monitoring gene expression is experimentally more tractable than comprehensively characterizing glycan structures associated with each model system, and we therefore chose this route for an initial investigation. While it is not possible to deduce glycan structures simply from gene expression of glycogenes, expression alterations in glycogenes provide insights on major structural alterations as well as leads on target points for therapeutic intervention. Expression alterations in glycogenes previously have been examined using focused microarrays or PCR arrays designed to specifically measure the relevant transcripts [32], [33], [34]. Here we demonstrate the use of whole-genome expression profiling to capture the same information by selectively analyzing data from a target gene list of 587 glycan-associated genes (including genes relevant to EMT). An advantage of this approach is the ability to use standard expression profiling platforms as well as historical data that was not generated specifically to examine glycogenes. We used cell culture systems in which EMT could be controlled by stimulation with TGFβ (model 1) or in which the terminal differentiation state of a variety of cell lines could be classified as either epithelial-like or mesenchymal-like (model 2). In addition, we examined gene expression patterns in cells that were either actively migrating or stationary (model 3). Each model system represents a different aspect of cell behavior relating to EMT: active transition between states, terminal differentiation states, or the activity of migration. We report here the major alterations to glycan-associated gene expression that are associated with each of the model systems and that are common between the model systems.

## Results

### Glycogene expression in TGFβ-induced EMT

The first model system in which we examined glycogene expression changes was TGFβ induction of EMT in the cell lines Panc-1 and A549. The A549 cell line was not derived from pancreatic cancer but was included to provide information about the generality of the associations with EMT. In addition, by looking for genes that change in both cell lines, we reduced the list of candidate genes. TGFβ treatment resulted in dissolution of cell-cell adhesion and increases in spindle-like projections in both cells lines (Figure S1). Whole-genome expression measurements were obtained for the treated and untreated cells using Affymetrix gene chips. For a focused analysis on genes of interest, we used our list of 587 genes related to glycosylation and EMT-associated pathways (see Table S1).

We first examined whether glycosylation-related genes showed a higher rate of expression changes than all other genes (Table 1), which may indicate a prominent role for glycosylation alterations in EMT. Among all genes, 1,524 unique Affymetrix targets changed expression in both Panc-1 and A549 after treatment with TGFβ. Since our glycan related genes represented 1.5% of the total unique Affymetrix U133 Plus 2.0 targets, random representation of glycogenes predicts a corresponding 1.5% representation of glycogenes (or 23 genes) among those that changed expression. However, 40 of the 1,524 genes (2.6%) were glycogenes from our target list. This difference was significant by chi-square analysis (X^2^ = 13.05, p<0.05). This result suggested an enrichment of transcriptional regulation of glycan-related genes in TGFβ-induced EMT.

**Table 1:** Rate of glycogene expression alterations.

	Model System		
	Induced EMT^1^	Cell line panel^2^	Migrating cells^3^
Total genes probed	35,888	35,888	16,087
Glycogenes probed (% of total)	555 (1.5%)	555 (1.5%)	525 (3.3%)
Total genes changing	1524	3675	2778
Glycogenes changing (% of total changing)	40 (2.6%)	87 (2.4%)	84 (3.0%)
Glycogene enrichment (p value, Chi-squared)	p<0.05	p<0.005	Not significant

1 Changed genes defined as significant (p<0.05) difference in the triplicate Panc-1 cells and +/−0.25 fold change in the A549 cells.

2 Changed genes defined as p<0.05 between mesenchymal-like and epithelial-like cell lines.

3 Changed genes defined as +/−0.25 fold change in both Panc-1 and MiaPaCa cells.

An examination of the genes that were altered more than 1.5-fold in both cell lines (Tables 2 and 3) revealed some functional themes. The greatest change was in the proteoglycan SPOCK1, which contains glycosaminoglycan side chains of heparin- and chondroitin-sulfate [35]. Related to this protein, genes that modify glycosaminoglycan sulfation were up-regulated, including SULF2, CHST3, and CHST11, and HAS2 was up-regulated, which is involved in the synthesis of the glycosaminoglycan hyaluronan. A lectin-like receptor involved in cell motility and extracellular matrix remodeling, MRC2, was upregulated. Alterations to the initiation of O-glycosylation were indicated by GALNT2 and GALNT10 changes, and matrix adhesion changes were represented by the upregulation of several integrins and the matrix protein LAMC2 (gamma 2 laminin). Other elevated genes represent EMT and TGF signaling functions. The six genes with reduced expression showed no particular enrichment in specificity, with ST8SIA4 being the only reduced glycosyltransferase.

**Table 2:** Genes with increased expression in TGFβ-induced EMT.

Category	Symbol	Name^1^	Entrez ID	EMT Induced Panc-1 Fold Change^2^	EMT Induced A549 Fold Change^2^
Glycoproteins	SPOCK1	sparc/osteonectin, cwcv and kazal-like domains proteoglycan (testican) 1	6695	8.04	70.53
TGFâ Pathway	LTBP2	latent transforming growth factor beta binding protein 2	4053	10.33	9.42
**Glycan Degradation**	**SULF2**	**sulfatase 2**	**55959**	**2.84**	**9.23**
**EMT Marker**	**ZEB1**	**zinc finger E-box binding homeobox 1**	**6935**	**8.31**	**2.74**
Glycoproteins	LAMC2	laminin, gamma 2	3918	2.92	6.45
Notch pathway	JAG1	jagged 1 precursor	182	5.54	3.10
Glycoproteins	ITGA2	integrin, alpha 2 (CD49B, alpha 2 subunit of VLA-2 receptor)	3673	3.24	5.13
Glycan-transferase	CHST3	carbohydrate (chondroitin 6) sulfotransferase 3	9469	2.31	4.30
TGFâ Pathway	TGFB1I1	transforming growth factor beta 1 induced transcript 1	7041	1.82	4.78
Glycoproteins	ITGB3	integrin, beta 3 (platelet glycoprotein IIIa, antigen CD61)	3690	3.84	2.58
Glycan-transferase	HAS2	hyaluronan synthase 2	3037	1.50	4.87
Glycan-transferase	CHST11	carbohydrate (chondroitin 4) sulfotransferase 11	50515	1.60	4.62
Glycoproteins	ITGA5	integrin, alpha 5 (fibronectin receptor, alpha polypeptide)	3678	1.70	3.74
TGFâ Pathway	TGFB1	transforming growth factor, beta 1	7040	2.61	2.57
Glycan-transferase	GALNT2	polypeptide N-acetylgalactosaminyltransferase 2	2590	2.35	2.78
Glycan-transferase	GALNT10	ppGalNAc T10; GalNAc transferase 10	55568	2.19	2.64
EMT Marker	ELK3	ETS-domain protein (SRF accessory protein 2)	2004	1.81	2.99
Nuc. Sugar	SLC35F2	solute carrier family 35 member F2	54733	1.83	2.74
Glycoproteins	ITGAV	integrin, alpha V (vitronectin receptor, alpha polypeptide, antigen CD51)	3685	1.58	2.68
CBP:C-Type Lectin	MRC2	endocytic receptor (macrophage mannose receptor family) 2mannose receptor, C type 2	9902	1.98	2.15
Glycoproteins	ITGA3	integrin, alpha 3 (antigen CD49C, alpha 3 subunit of VLA-3 receptor)	3675	2.08	1.90

**Table 3:** Genes with decreased expression in TGFβ-induced EMT.

Category	Symbol	Name^1^	Entrez ID	EMT Induced Panc-1 Fold Change^2^	EMT Induced A549 Fold Change^2^
**Glycoproteins**	**CDH1**	**cadherin 1, type 1, E-cadherin (epithelial)**	**999**	**4.01**	**9.52**
Glycan-transferase	ST8SIA4	ST8 alpha-N-acetyl-neuraminide alpha-2,8-sialyltransferase 4	7903	2.96	8.16
CBP:C-Type Lectin	KLRC3	killer cell lectin-like receptor subfamily C, member 3	3823	6.84	2.78
CBP:C-Type Lectin	CD302	C-type lectin domain family 13, member A	9936	2.31	3.13
EMT Marker	TWIST2	twist homolog 2 (Drosophila)	117581	2.66	2.27
Galectin	LGALS3BP	Galectin 6 binding protein	3959	1.91	2.78

1 Only probes showing a greater than +/−50% change in expression were included. Genes in bold show a common trend with the 22-cell line panel.

2 The results were averaged when multiple probes for a given gene were present.

### Glycogene Expression in a Pancreatic Cancer Cell Line Panel

**Figure 1:**
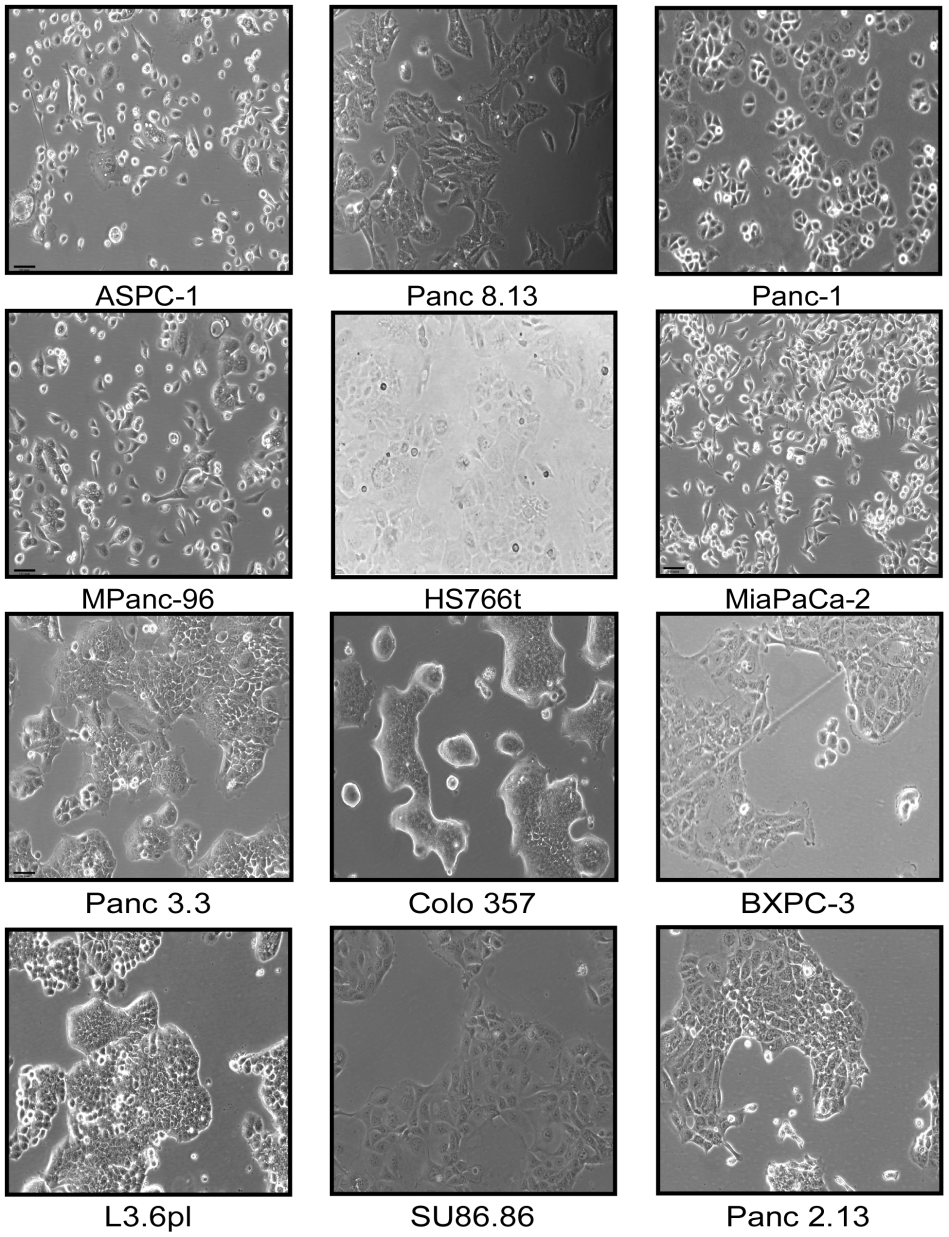
Morphologies and clustering of selected pancreatic cancer cell lines. Mesenchymal-like cell lines are presented in the top two rows, and epithelial-like cell lines are in the bottom two rows. Note the increased cell scatter and spindle like projections seen in the mesenchymal-like cells as compared to the tighter cell-cell adhesion and more spherical shape characteristic of the epithelial cells. The images were collected by phase-contrast microscopy at 10× magnification.

The second model system was a panel of 22 pancreatic cancer cell lines. A diversity of cell morphologies and adhesion behaviors was observed among the cell lines (Figure 1). Certain cell lines have strong epithelial characteristics (rounded morphologies and numerous cell-cell contacts) while others are clearly mesenchymal-like (spindle-like projections and few cell-cell adhesions). Therefore we sought to identify gene expression characteristics that differentiate these two behaviors. This comparison does not directly address EMT, but rather gives insights into the terminal differentiation states associated with the mesenchymal and epithelial phenotypes.

**Figure 2:**
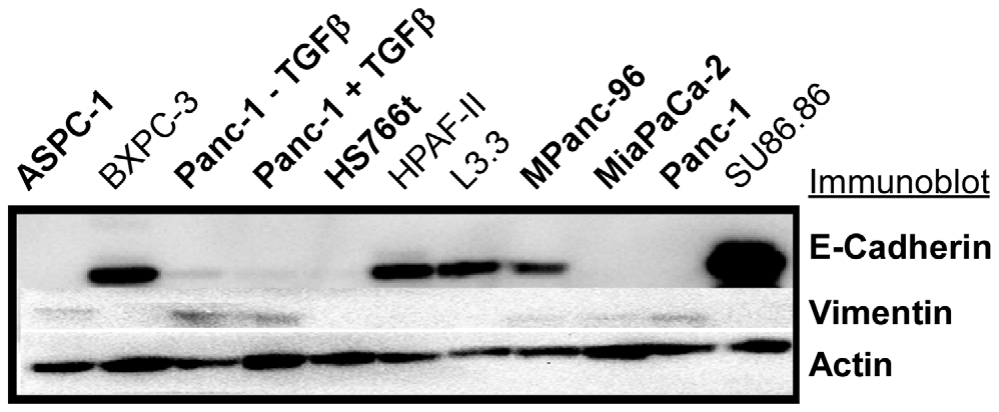
E-cadherin and vimentin protein levels in selected pancreatic cancer cells. Lysates were collected from the selected cell lines, fractionated by SDS-PAGE, and probed by Western blot. The highlighted bands are at the expected molecular weights of 110 kD for E-cadherin, 57 kD for vimentin, and 42 kD for actin.

We initially classified the cell lines as either epithelial-like or mesenchymal-like based on morphology and clustering (Figure 1). Cell lines such as CAPAN-2, HPAF-II, and BXPC-3 have dense, rounded colonies and were unambiguously classified as epithelial-like. On the other end of the spectrum MiaPaCa-2 and MPanc-96 clearly showed the mesenchymal characteristics of low cell-cell interactions and spindle like cellular projections. Other cell lines were more difficult to classify by these characteristics, showing mixed or partial characteristics of each type. We therefore looked at the expression of EMT-related genes as a means of classification (Figure S2). Among those genes (see Figure S2 for the complete list) the ZEB1 transcription factor most clearly corresponded to morphology and strongly correlated with down regulation of the epithelial marker, E-cadherin (CDH-1) [6], [7], [8], [9] in the mesenchymal-like cells. A striking dichotomy of cells that were negative or positive for ZEB1 was evident, dividing the cell lines into six that were mesenchymal-like and 16 that were epithelial like. Based on this classification, the expression of E-cadherin (CDH-1) was significantly up regulated (p≤0.01) and the expression of vimentin (VIM) was significantly down regulated (p≤0.01) in the epithelial cells. The protein expression pattern of these two genes was confirmed by Western blot (Figure 2). Therefore we used this classification in subsequent analyses.

We examined whether glycan-related genes were different between the groups at a higher rate than all other genes (Table 1). Strictly by chance, since our glycan related genes represented 1.5% of the total unique Affymetrix U133 Plus 2.0 targets, we expected a corresponding 1.5% representation of glycogenes among the list of total changing genes. However, of the 3,675 probes that showed significantly altered levels of expression (p≤0.05) between the mesenchymal-like cells and the epithelial-like cells, 87 (2.37%) were glycogenes from our target list. A chi-square analysis showed these results to be highly significant (X^2^ = 18.9, p≤0.005). This trend continued at higher levels of significance as 1212 unique Affymetrix targets showed significantly different expression levels (p≤0.01). Of those targets, 30 (2.48%) were on our list as glycogenes, also a significant difference by Chi-square analysis (X^2^ = 8.12; p≤0.005). This analysis suggested an enrichment of transcriptional regulation of glycan-related genes in the differentiation of cells between mesenchymal and epithelial phenotypes.

**Figure 3:**
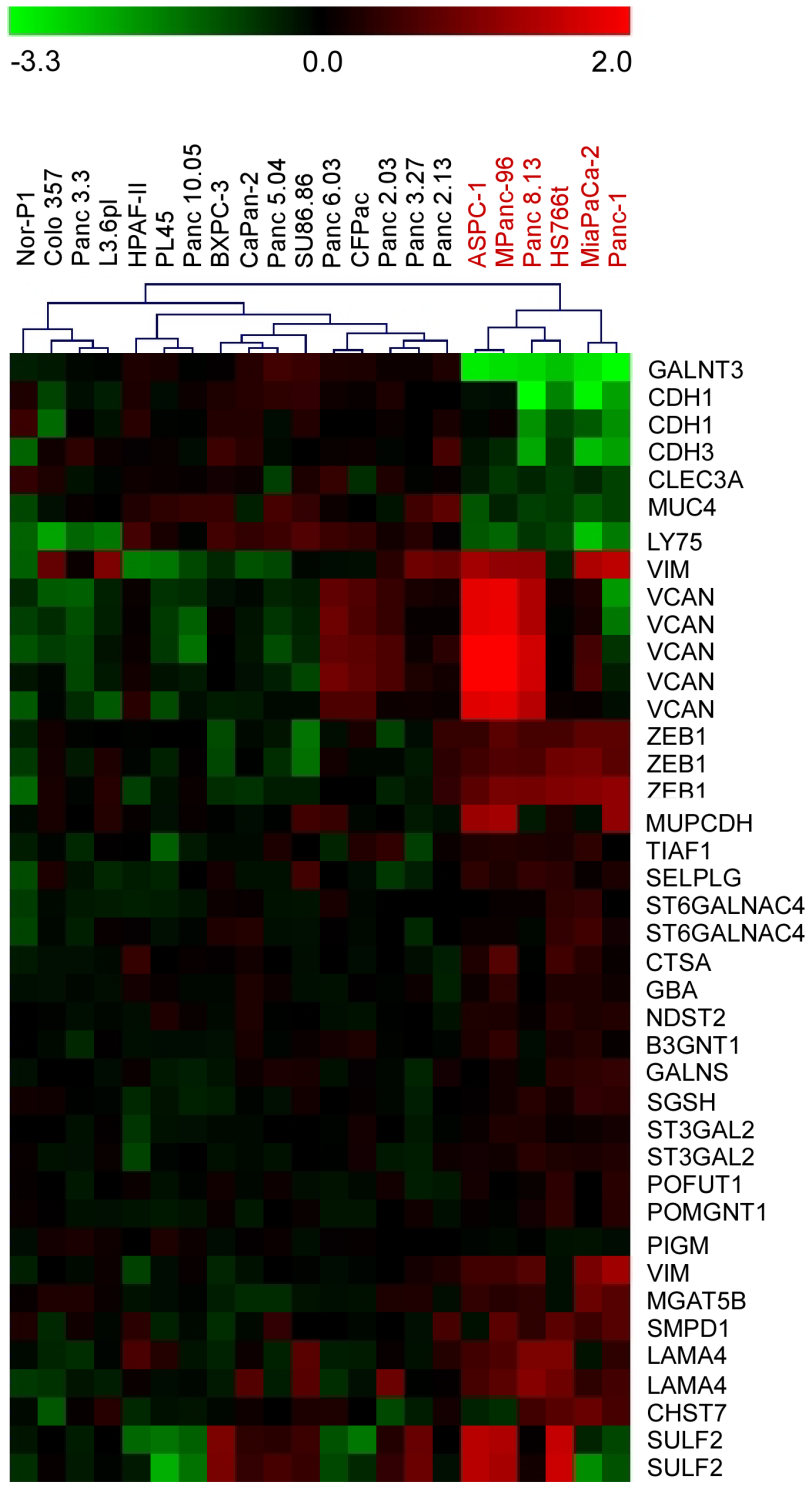
Expression patterns of genes discriminating the cell lines. Genes were included that had significantly different (p<0.01) expression levels between the mesenchymal-like (column labels colored red) and the epithelial-like (column labels colored black) cell lines. The expression values were log transformed (base 10) and median centered by row. The value of each square is indicated by the color bar.

The gene expression alterations for this model system are summarized in Tables 4 and 5. Similar to above, a proteoglycan was the most significantly overexpressed in the mesenchymal-like cells, in this case VCAN (versican), and genes regulating the sulfation of glycosaminoglycans were up-regulated, including SULF2, CHST7, SGSH, and NDST2. Matrix modification was also represented by upregulation of VIM (vimentin) and LAMA4 (alpha 4 laminin). Unlike in the induced-EMT model, genes involved in branching or extending glycan chains were up-regulated in the mesenchymal-like cells, including MGAT5B, ST3GAL2, ST6GALNAC4, POMGNT1, and B3GNT1. The mesenchymal-like cells also showed a striking down-regulation in some genes, most notably GALNT3, which initiates O-glycosylation. GALNT3 was down-regulated in all six mesenchymal-like cells, with an average decrease of ∼1000-fold. Two C-type lectins also were down-regulated, LY75 and CLEC3A. The expression patterns of these genes clearly distinguish the mesenchymal-like cell lines from the epithelial-like (Figure 3).

**Table 4:** Genes with higher expression in mesenchymal-like cell lines as compared to epithelial-like.

Category	Symbol	Name^1^	Entrez ID	p value^2^	Fold Change^2^
CBP:C-Type Lectin	VCAN	Versican	1462	2.81E-03 to 4.16E-03	20.75 to 39.03
**EMT Marker**	**ZEB1**	**zinc finger E-box binding homeobox 1**	**6935**	**1.55E-10 to 7.44E-08**	**4.98 to 11.72**
**Glycan Degradation**	**SULF2**	**sulfatase 2**	**55959**	**3.92E-03 to 5.77E-03**	**7.56 to 8.26**
EMT Marker	VIM	Vimentin	7431	4.61E-05 to 1.44E-03	7.38 to 7.55
Glycoproteins	MUPCDH	mucin and cadherin-like	53841	2.04E-03	7.14
Glycoproteins	LAMA4	laminin, alpha 4	3910	2.85E-03 to 2.90E-03	3.70 to 3.87
Glycan-transferase	CHST7	carbohydrate (N-acetylglucosamine 6-O) sulfotransferase 7	56548	2.61E-03	3.43
Glycan-transferase	MGAT5B	mannosyl (alpha-1,6-)-glycoprotein beta-1,6-N-acetyl-glucosaminyltransferase, isozyme B	146664	1.16E-03	3.35
Glycan Degradation	SMPD1	acid sphingomyelinase	6609	7.33E-04	2.97
Glycan Degradation	CTSA	cathepsin A precursor	5476	3.45E-03	2.39
Glycoproteins	SELPLG	selectin P ligand	6404	6.93E-03	2.24
Glycan Degradation	SGSH	N-sulfoglucosamine sulfohydrolase (sulfamidase)	6448	1.51E-04	2.10
Glycan-transferase	ST3Gal2	ST3 beta-galactoside alpha-2,3-sialyltransferase 2	6483	3.12E-05	2.05
Glycan-transferase	ST6GalNAc4	ST6 (alpha-N-acetyl-neuraminyl-2,3-beta-galactosyl-1,3)-N-acetylgalactosaminide alpha-2,6-sialyltransferase 4	27090	3.73E-03 to 8.64E-03	1.97 to 2.00
TGFâ Pathway	TIAF1	TGFB1-induced anti-apoptotic factor 1	9220	1.00E-02	1.93
Glycan Degradation	GALNS	N-acetylgalactosamine-6-sulfatase precursor	2588	4.85E-03	1.85
Glycan-transferase	NDST2	N-deacetylase/N-sulfotransferase (heparan glucosaminyl) 2	8509	1.85E-04	1.81
Glycan-transferase	POMGNT1	beta1,2-N-acetylglucosaminyltransferase	55624	3.70E-03	1.78
Glycan-transferase	POFUT1	protein O-fucosyltransferase 1	23509	4.00E-03	1.70
Glycan Degradation	GBA	glucosidase, beta; acid	2629	2.82E-03	1.70
Glycan-transferase	B3GNT1	UDP-GlcNAc:betaGal beta-1,3-N-acetylglucosaminyltransferase 1	11041	9.61E-03	1.64

**Table 5:** Genes with lower expression in mesenchymal-like cell lines as compared to epithelial-like.

Category	Symbol	Name^1^	Entrez ID	p value^2^	Fold Change^2^
Glycan-transferase	GALNT3	polypeptide N-acetylgalactosaminyltransferase 3	2591	2.17E-04	1116.29
CBP:C-Type Lectin	LY75	lymphocyte antigen 75	4065	7.06E-03	22.82
Glycoproteins	MUC4	Mucin 4, cell surface associated	4585	8.31E-03	11.55
Glycoproteins	CDH3	cadherin 3, P-cadherin (placental)	1001	4.49E-03	7.98
**Glycoproteins**	**CDH1**	**cadherin 1, type 1, E-cadherin (epithelial)**	**999**	**9.91E-04 to 9.74E-03**	**3.38 to 5.78**
CBP:C-Type Lectin	CLEC3A	C-type lectin domain family 3 member A	10143	2.21E-03	4.76
Glycan-transferase	PIGM	phosphatidylinositol glycan anchor biosynthesis	93183	7.42E-03	1.57

1 Only genes showing a significant change (p≤0.01) and greater than +/−50% change in expression were included. Genes in bold show a common trend with the EMT induced cancer cell lines.

2 Genes represented by multiple probes have p values and fold changes represented as ranges.

### Glycogene expression in migrating and stationary pancreatic cancer cells

The third model system was a comparison of actively migrating cells to stationary cells [36]. Panc-1 and MiaPaCa-2 cells were seeded as 1 mm defined circumference and cells were allowed to migrate for 48 h. Two distinct morphological populations were observed. The cells on the periphery of the cell cluster migrated away from the region of high cell density (rim) and took on a classical mesenchymal phenotype with spindle like projections and loss of cell to cell adhesion. Cells that remained in the highly populated center (core) showed greater intercellular adhesion and were more rounded in appearance. The cells were harvested into “rim” and “core” groups by selectively collecting from each of these two groups.

2,778 unique targets changed expression at least +/−0.25-fold in both the migrating MiaPaCa-2 and Panc-1 when compared to their stationary counterparts (Table 1). Since our glycan-related genes represented 3.0% of the total unique Agilent Whole Human Genome Microarray Kit (4×44K probes), random chance predicted a corresponding 3.0% representation of glycogenes among the list of genes that changed. That level of representation was indeed observed, as 84 of the 2778 genes (3.0%) with altered levels of expression were glycogenes from our target list. A chi-square analysis revealed that the difference between the observed change in glycogenes was not significant compared to the expected value. These results do not suggest an enrichment of regulation of glycan-related genes in characterizing differences between actively migrating and stationary cells.

Fewer genes showed differential expression between the groups than in the above models (Tables 6 and 7). Eight target genes increased expression by more than 1.5-fold in both migrating MiaPaCa-2 and Panc-1. No genes increased by more than two-fold in both cell types. No clear themes in glycogene function were discernable among the genes with increased expression. Nine target genes decreased expression levels over 1.5 fold in both Migrating MiaPaCa-2 and Panc-1. Two are related to the removal of mannose in the biosynthesis of N-glycans, MAN1A2 and MANBA. The mucin MUC12 was the only gene with decreased expression greater than two-fold in both cell types.

**Table 6:** Genes with higher expression in cell migration.

Category	Symbol	Name^1^	Entrez ID	Panc-1 Migrate Fold Change^2^	MiaPaCa-2 Migrate Fold Change^2^
Glycan-transferase	Gal3ST3	galactose-3-O-sulfotransferase 3	89792	3.88	1.54
Notch Pathway	Notch4	Notch homolog 4	4855	2.93	1.83
Glycan-transferase	FUT4	fucosyltransferase 4 (alpha (1,3) fucosyltransferase, myeloid-specific)	2526	1.80	2.12
Glycan-transferase	GALNT13	ppGalNAc T13; UDP-N-acetyl-alpha-D-galactosamine:polypeptide	114805	2.28	1.61
Glycoproteins	MCOLN1	mucolipin 1	57192	1.80	1.84
Glycoproteins	CDH15	cadherin 15, type 1, M-cadherin (myotubule)	1013	1.68	1.93
Glycoproteins	ITGA2	integrin, alpha 2 (CD49B, alpha 2 subunit of VLA-2 receptor)	3673	1.94	1.50
CBP:C-Type Lectin	REG4	regenerating islet-derived family, member 4	83998	1.51	1.64

**Table 7:** Genes with lower expression in cell migration.

Category	Symbol	Name^1^	Entrez ID	Panc-1 Migrate Fold Change^2^	MiaPaCa-2 Migrate Fold Change^2^
Glycoproteins	MUC12	mucin 12, cell surface associated	10071	2.31	3.45
Glycan Degradation	MAN1A2	mannosidase alpha class 1A member 2	10905	1.89	2.59
TGFβ Pathway	TGFBR3	transforming growth factor, beta receptor III	7049	1.79	2.50
Nuc. Sugar	SLC35A3	solute carrier family 35 (UDP-N-acetylglucosamine (UDP-GlcNAc) transporter), member A3	23443	1.88	2.33
Glycan Degradation	GALC	galactosylceramidase precursor	2581	1.53	1.92
Glycan-transferase	CHST1	carbohydrate (keratan sulfate Gal-6) sulfotransferase 1	8534	1.83	1.59
CBP:C-Type Lectin	ATRN	Attractin	8455	1.59	1.82
Glycan Degradation	MANBA	mannosidase, beta A, lysosomal	4126	1.57	1.56
CBP:C-Type Lectin	REG3A	Pancreatitis-associated protein	5068	1.54	1.54

1 Only probes showing a greater than +/−50% change in expression for both cell lines were included.

2 Genes represented by more than one probe are represented as averages.

### Shared Genes and Functional Themes

We next investigated whether certain gene expression changes were common between two or more of the model systems. Such an analysis is useful to provide more guidance on which genes are important in multiple aspects of EMT or are functionally important in processes related to EMT. The overlap between the model systems was slight. In comparing TGFβ-induced EMT and the cell line panel, only three genes were common: ZEB1 and SULF2 were up-regulated in both models, and CDH1 was decreased in both. Using data only from the Panc-1 cell line in the TGFβ-induced and migrating cell models, ST6GALNAC4 increased and PIGM decreased in all model systems. These results show that while glycogenes are altered in each model and are particularly enriched in induced-EMT and the cell line panel, the specific genes involved in each are divergent between the systems.

**Figure 4:**
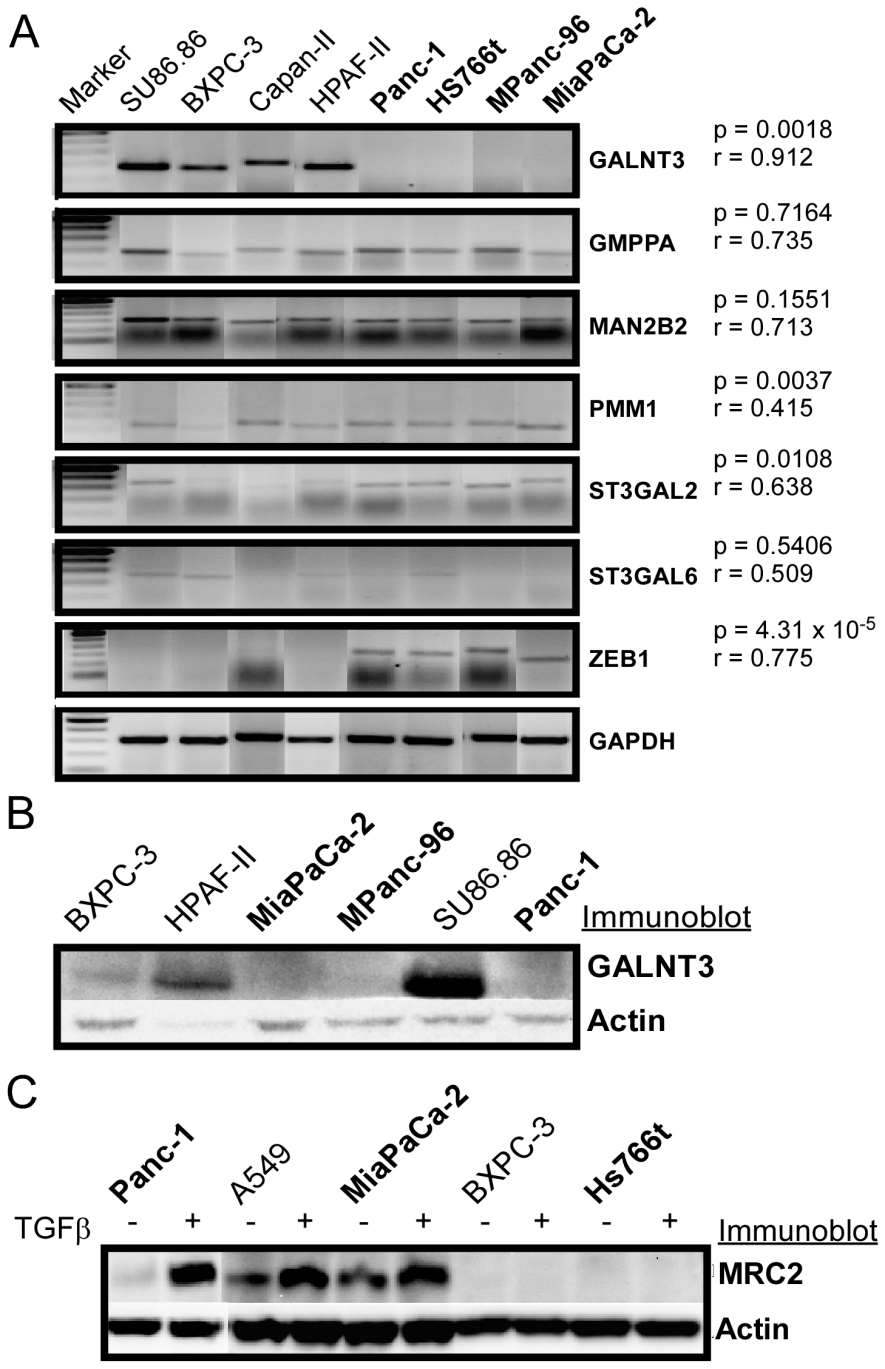
RT-PCR and Western blot verification of microarray data for selected pancreatic cancer cell lines. (A) RT-PCR was performed on lysates from the indicated cell lines, and intensities of the bands at the expected size for each gene were quantified. A student's t-test (results given by the p value) was performed comparing the band intensities between the mesenchymal-like cell lines (in bold) and the epithelial cell lines. In addition, a correlation was calculated (results given by the r value) between the RT-PCR results and the microarray data. Glyceraldehyde-3-phosphate dehydrogenase (GAPDH) was used as a cDNA loading control. (B) Comparison of GALNT3 protein levels by Western blot in selected pancreatic cancer cell lines. Lysates were collected from the selected cell lines, fractionated by SDS-PAGE, and probed by Western blot. Mesenchymal-like cell are labeled in bold. The highlighted bands are at the expected molecular weights of 73 kD for GALNT3 and 42 kD for actin. (C) MRC2 protein levels measured by Western blot in TGFβ treated and untreated cells. The cell lines were either exposed to 5 ng/mL TGFβ or untreated under serum starvation for 72 hours prior to cell lysis. The highlighted bands are at the expected molecular weights of ∼167 kD for MRC2 and 42 kD for actin.

Although many of the altered genes were different between the induced-EMT model and cell line panel, several functional themes were present in both. The most predominant shared theme was the modulation of the glycosaminoglycan (GAG) component of the extracellular matrix. SPOCK1 and VCAN (versican) are both matrix proteins bearing GAG chains, and both systems showed up-regulation of genes that add and take away sulfate from those chains, including SULF2, CHST3, CHST11, HAS2, CHST7, SGSH, and NDST2. An Alcian Blue assay was conducted to further characterize the overall sulfation levels between the epithelial and mesenchymal-like cell lines as well as after induction of EMT in Panc-1 by TGFβ (Figure 4). A significant increase in overall sulfation was seen for the mesenchymal-like cell lines (p = 1.29×10^−5^). Inducing EMT in Panc-1 by TGFβ continued the trend of significantly increasing overall levels of sulfation (p = 0.027). These results confirm cellular sulfation changes for the two model systems in which they were suggested by gene expression.

Another theme in common between the induced-EMT model and the cell line panel was alterations to lectin-like receptors of the mannose receptor family. The four members of this family, MRC1, MRC2, LY75, and CD302, are transmembrane endocytic receptors with multiple carbohydrate-binding domains. MRC2 was upregulated and CD302 was down-regulated in the induced-EMT model, and LY75 was down-regulated in the cell line panel. The two EMT models also shared alterations to member of the N-acetyl galactosamine (GalNAc) transferase family, which catalyze the initial addition of GalNAc to a serine or threonine residue in mucin-type O-linked glycosylation. GALNT2 and GALNT10 were up-regulated in induced EMT, and GALNT3 was the strongest discriminator of epithelial-like and mesenchymal-like cells in the cell line panel, with expression dropping below measureable levels in the mesenchymal cells (Figure 4).

### Validation of Gene Expression Differences

**Figure 5:**
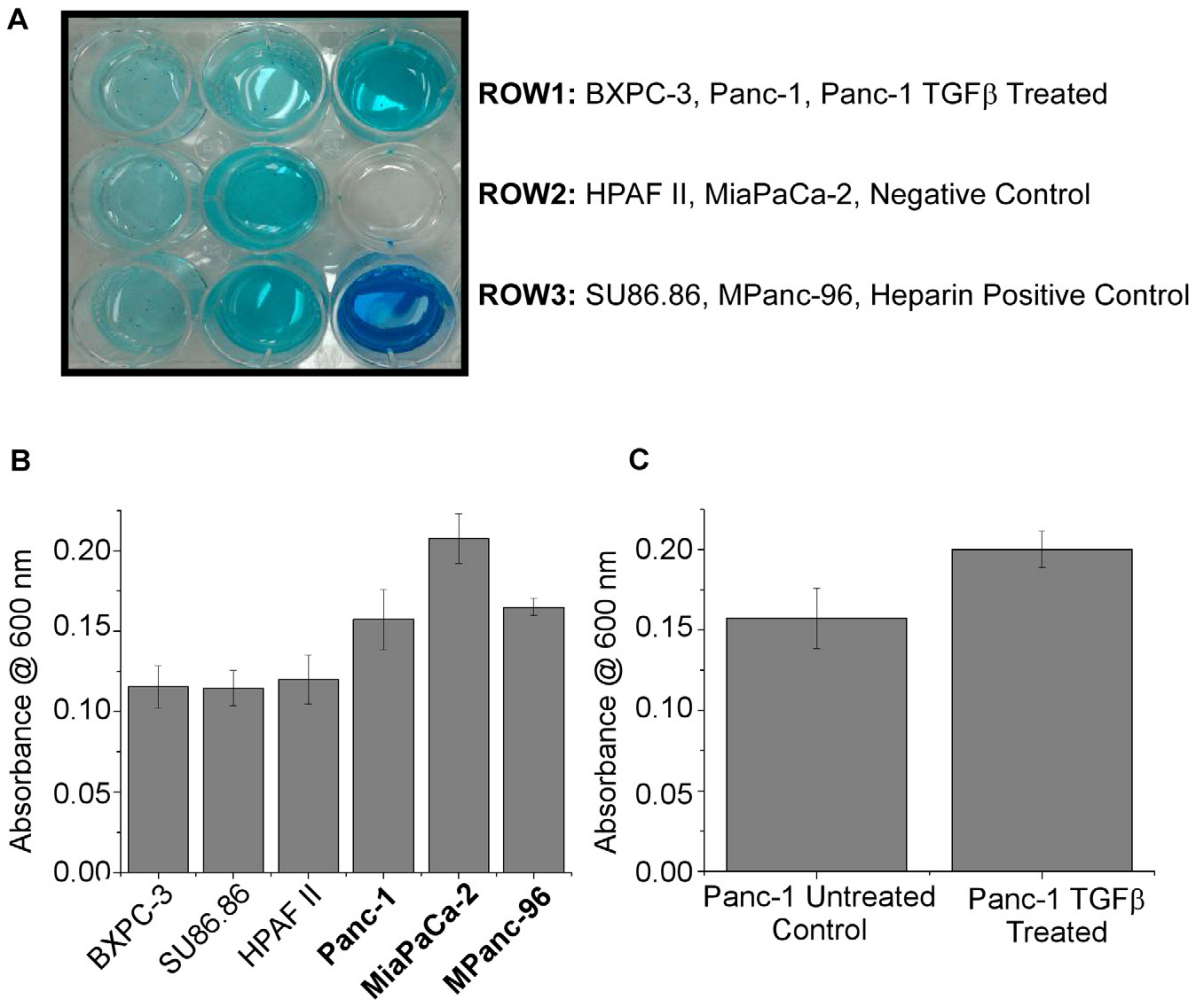
Alcian Blue assay for comparison of overall sulfation levels for selected pancreatic cancer cell lines. An Alcian Blue assay was performed on lysates from the indicated cell lines, and the overall sulfation for each cell line was quantified by measuring the absorbance of the dye at 600 nm. A student's t-test (results given by the p value) was performed comparing the absorbances between the mesenchymal-like cell lines (in bold) and epithelial cell lines as well as between Panc-1 after 72 hours of TGFβ treatment and untreated Panc-1. Heparin was used as a sulfated GAG control. (A) Photograph of the resulting retention of Alcian Blue dye after the precipitation of sulfated glycans and subsequent re-suspension. The second column represents the mesenchymal-like cell lines and retained more stain, indicating the presence of higher levels of sulfated glycans in the cell lysates. (B) Bar graphs showing the differences in absorbance at 600 nm for the cell lines. The mesenchymal-like cell lines (in bold) had significantly higher levels of absorbance than the remaining epithelial cell lines (p = 1.29×10^−5^). (C) Bar graphs showing the differences in absorbance at 600 nm for Panc-1 after 72 hours exposure to 5 ng/mL TGFβ or untreated under serum starvation. Panc-1 after TGFβ treatment had significantly higher levels of absorbance than the untreated Panc-1 (p = 0.027).

The differential gene expression in the mesenchymal-like and epithelial-like cell lines was confirmed for certain genes using RT-PCR and Western Blot (Figure 5). The correlations between the RT-PCR and microarray results were generally consistent, with the strongest correlations seen for GALNT3, 0.912; ZEB1, 0.774; GMPPA, 0.735; and MAN2B2, 0.713; and weaker correlations for ST3GAL6, 0.509; and PMM1, 0.415. Protein-level changes were confirmed by Western Blot for GALNT3 and MRC2. GALNT3 detection was positive for the epithelial cell lines BXPC-3, HPAF II, and SU86.86 while remaining undetected in the mesenchymal-like cell lines MiaPaCa-2, MPanc-96, and Panc-1. MRC2 was only detected in A549, Panc-1, and MiaPaCa-2 and showed increased levels after treatment with TGFβ in these three cell lines. There was no detectable level of MRC2 for BXPC-3 or HS766t in the presence or absence of TGFβ.

## Discussion

The ability to detect or control EMT in pancreatic cancer could lead to improved treatment strategies. The characterization of molecular alterations associated with EMT is a first step in unraveling some of the mechanisms driving this process. Defining the glycan changes associated with EMT may be especially important for understanding this process, given the known involvement of glycans in other cell differentiation processes. Whole-genome expression profiling combined with a target gene list of 587 glycosylation-related genes was a practical means of investigating glycosylation in this biological system. In addition, the use of three in-vitro model systems proved useful for examining complementary aspects of the epithelial and mesenchymal states, namely the active transition between states, the terminal differentiation states, and the act of migration.

The importance of glycan alterations in EMT was supported by the frequency of changes to glycosylation-associated genes (Table 1). Glycan-associated genes were altered more frequently than was predicted in two of the model systems, TGFβ-induced EMT and the cell line panels, but not in the cell migration model. The finding that glycan remodeling is a major aspect of the cell differentiation process would be consistent with previous work showing the importance of glycan alterations in stem cell biology and immune activation [27], [31], [37]. The lack of major glycogene changes in cell migration between may indicate that the migrating cells were not actually differentiating, but rather just altering expression for the mechanics of migration. Therefore glycan changes may be more important as indicators of cell type and status rather than as functional contributors to migration. However, it should be noted that alteration of glycosyltransferase activity in cell line models have shown differential effects on migratory ability *in vitro* [38], [39], [40]. In the case of Panc-1 and MiaPaCa-2, they may already possess the glycan-machinery necessary for an enhanced migratory ability; further transformation may not be necessary. This area of the investigation was also limited to two pancreatic cancer cell lines in very specific *in vitro* conditions. Future analyses using more cell lines and different methods of separating migratory from stationary cells may yield new findings.

The functional themes shared by the induced EMT model and the cell line panel give some insights into the roles of glycosylation in EMT. The alterations of proteoglycans and sulfation enzymes suggest the importance of the control of GAG sulfation in EMT. This finding is consistent with previous research showing alterations to matrix components such as versican [41] and alterations to GAG sulfation [42] in association with cancer, particularly in aggressive forms of cancer. The cancer-promoting functions of versican may act through regulating growth factor activity and by interacting with immune and stromal cells [43], functions which could be modified by alterations to sulfation. SPOCK1 may function through similar mechanisms but is less well studied. SULF2, which was strongly up-regulated in both systems and acts to remove sulfates from certain GAG chains, also has been associated with lung carcinogenesis [44] and the tumorigenicity of pancreatic cancer cells [45]. The cancer-promoting functions of SULF2 appear to work through activating Wnt signaling or releasing growth factors from the extracellular matrix, which may also have a pro-angiogenic effect. The association of these functions with cancer EMT suggests that cancer cells undergoing EMT use a reconditioning of the extracellular space to become highly invasive. If so, interfering with GAG sulfation and desulfation, or the interactions induced by modified sulfation, represent attractive targets for intervention, particularly since these molecules reside in the extracellular space.

The evaluation of total sulation levels using Alcian Blue staining revealed an increase in overall sulfation for the mesenchymal-like cell lines as well as an increase in Panc-1 sulfation after treatment with TGFβ. This effect could be due to an increase in GAG-bearing proteins such as SPOCK1 or VCAN, as seen in the induced EMT and mesenchymal-like cell line panel, respectively. Another possibility is increased sulfate on each GAG chain, which could be induced by increased sulfotransferase levels. We observed higher levels of CHST3 & CHST11 in the induced EMT model and higher CHST7 & NDST2 in the mesenchymal-like cell panel. These pro-sulfation activities must outweigh the effects of the increased SULF2 sulfatase, which removes sulfates. Since SULF2 is specific for heparin and heparan sulfate [46], and CHST3, CHST11, and CHST7 act on chondroitin sulfate, an interesting possibility is a shift in sulfation from heparan sulfate to chondroitin sulfate. Further researh will address the nature of the increased sulfation levels as well as the biological effects of these alterations.

The alterations in mannose receptor family members could have implications for cellular interactions with a reconditioned local environment. Mannose receptor family members have specificity for various matrix components such as collagen and sulfation [47]. Collagen was up-regulated in the media of both A549 and Panc-1 as determined by mass-spectrometry profiling (data not shown), and multiple other matrix components are altered by cancer cells, as discussed above. Considering these factors, altered regulation of mannose receptors such as MRC2 could be a means to conditioning the cellular response to the new matrix environment or acting to mediate autocrine loops. In addition, MRC2 is involved in chemotaxis and invasion [48] and could be important for the acquisition of those traits by cancer cells.

UDP-N-acetyl-d-galactosamine: polypeptide UDP-N-acetylgalactosaminyltransferases (GALNTs) initiate O-glycosylation by attaching a GalNAc to a serine or threonine on target proteins. There are currently 15 reported isozymes in the GALNT family with variations in substrate specificity [20]. Many of the GALNTs are ubiquitously expressed in human tissue, but several show restricted tissue expression [49], [50]. This diversity allows variation in the glycoforms of various proteins depending upon cell origin and cell state [51]. Alterations to O-glycan initiation through modulation of the GALNTs, particularly GALNT3, may also affect cancer cell behavior by changing the glycoform of target protein(s) and thereby altering interactions with the cellular environment. Notably, GALNT3 expression is restricted to glandular epithelial cells in normal tissue [49] and adenocarcinomas [52]. Consistent with our observations here, loss of GALNT3 previously has been associated with the mesenchymal phenotype [51] as well as with a poorer cancer prognosis [52], [53], [54]. The identification of the particular proteins acted upon by GALNT3 will be a future research goal.

The genes that were common between the model systems may be of particular interest for further study. Changes in the expression of ST6GALNAC4 were shared between all pancreatic cell experiments (excluding the A549 lung cancer cell line). ST6GALNAC4 catalyzes the addition of sialic acid (Neu5Ac) to the 6′ carbon of N-acetylgalactosamine (GalNAc) of the acceptor motif Neu5Ac-alpha-2,3-Gal-beta-1,3-GalNAc on glycoproteins and glycolipids, resulting in a terminal, di-sialylated glycan structures [20]. It was noteworthy that a functionally-related gene, ST3GAL2, was also up-regulated in the mesenchymal-like cells of the cell line panel. ST3GAL2 catalyzes the addition of Neu5Ac in alpha 2,3 linkage to galactose in the acceptor motif Gal-beta-1,3-GalNAc [20], [55], potentially forming the acceptor motif for ST6GALNAC4. Cancer-associated glycans have been shown to have increased sialylation, resulting in increased metastatic potential [50], [56] and tumor growth [57]. An unexpected result was seen in the induced EMT model for a sialyltransferase: ST8 alpha-N-acetyl-neuraminide alpha-2,8-sialyltransferase 4 (ST8SIA4). This enzyme is one of several family members capable of forming polysialic acid (PSA), a known modulator of neural cell adhesion via the neural cell adhesion molecule (NCAM) [58]. Investigations into the role of PSA in pancreatic cancer has shown that PSA-NCAM represses E-cadherin mediated cell-cell adhesion and that removal of PSA results in increased cell aggregation and reduced migration [59]. Loss of ST8SIA4 would then be expected to favor a decrease in migration. However, it should also be noted that NCAM was not detected in our study at the transcript level (data not shown).

The second gene expression change shared between all pancreatic experiments was down-regulation of PIGM (phosphatidylinositol glycan anchor biosynthesis, class M). PIGM catalyzes the initial mannosylation in the formation of glycosylphosphatidylinositol (GPI) membrane anchors. GPIs are glycolipids that are involved anchorage dependent tethering of many cell surface proteins [60]. Decreased expression of PIGM due to promoter mutation has been shown to cause severe GPI anchor deficiency [61]. A decrease in PIGM levels due to downregulation of the gene might also lead to a decreased prevalence of GPI anchored proteins at the cell surface.

These insights into the genes and glycosylation patterns that are associated with the development and maintenance of mesenchymal-like pancreatic cancer cells can be applied to future studies in a variety of ways. Future work to characterize the structures of the glycans using mass spectrometry should be compared to these gene expression results to piece together the links between the biosynthetic pathways and the most prevalent glycan alterations in EMT. If these molecular alterations prove to be highly selective to mesenchymal-like cancer cells, therapeutic strategies based on this information may be highly effective to particularly target the cells most involved in cancer progression and drug resistance. Methods to selectively modulate glycosylation or protein-glycan interactions at key points have been investigated for inflammation, metastasis, and pathogen invasion [62], [63], [64], [65]. The information from this study also may be useful to develop biomarkers to detect mesenchymal-like cancer cells. We previously showed that measuring glycans on specific proteins can produce improved biomarker performance over just measuring protein abundances, due to the fact that both protein abundance and glycosylation can be altered in cancer [66], [67]. Future studies will aim to identify the proteins that enzymes identified here act upon, so that the glycosylation levels on those proteins can be efficiently probed using antibody-lectin sandwich arrays (ALSA) [66], [67]. Using high-throughput antibody array methods developed earlier, we can rapidly characterize the associations between various glycoforms and clinical states.

This study provides an initial look into the nature of glycan alterations in cancer EMT and supports the concept of the importance of glycans in both the transition to and the maintenance of the mesenchymal state. Alterations to matrix components, the sulfation of GAGs, mannose receptors, O-glycosylation, and specific sialylated structures characterize pancreatic cancer EMT. The specific genes controlling these modifications may be targets to prevent or reverse EMT, and the glycan structures produced by these genes, coupled with the proteins on which they are found, may provide molecular hooks to specifically detect mesenchymal-like cancer cells. Glycan structural analyses will provide further details about the activities of these genes. These findings provide specific leads for future research into the detection and targeting of particularly aggressive or drug-resistant forms of pancreatic cancer cells.

## Materials and Methods

### Cell Culture and RNA Extraction

The cell lines used in the TGFβ induced EMT and migration models were purchased from the American Type Culture Collection (ATCC, Manassas, VA). The 22 cell lines used in the pancreatic cancer cell line panel were generously provided by Drs. Martin McMahan and Stephan Gysin (University of California San Francisco). All cells were grown according to the ATCC recommended culture conditions for each cell line. For the induced EMT model, Panc-1 and A549 were grown to 70% confluency, cultured for one day in serum-free conditions, and incubated for 48 hours with fresh media containing either 5 nM TGFβ (R&D Systems), or plain media. The cells were rinsed three times in 1× phosphate-buffered saline prior to lysis by the addition of TRIzol reagent (Invitrogen, Carlsbad, CA) followed by repeated passes through an 18-G needle and a 5 min incubation at RT. The lysates were vigorously mixed with chloroform and spun down at 12000×g for 20 minutes at 4°C. The aqueous layers were transferred to an isopropanol solution, mixed vigorously, and centrifuged at 12000×g for 30 minutes at 4°C. The RNA-containing pellet was washed with 75% ethanol, dried, and dissolved in RNase-free water. The RNA quantity and quality was determined using a spectrophotometer (Bio-photometer, Eppendorf, Westbury, NY) and stored at −20°C until all cell lines were ready for cDNA synthesis and polymerase chain reaction (PCR). For the cell line panel model, all cells were grown to 70% confluency under normal culture conditions prior to lysis and RNA extraction using the above protocol.

The radial migration assay was preformed as described previously [68]. Isolation of sub-populations of stationary and migratory cells were performed as described previously [36]. Briefly, to simulate a migratory front (rim) and proliferating core, five thousand pancreatic cells were seeded as a confluent, circular monolayer using cell sedimentation manifolds (CSM, Inc., Phoenix, AZ) on Collagen IV-coated, 10-well slides. Migration was initiated by removing the manifolds 16 hours after seeding, and the cells were allowed to radially disperse for 24 hours. Stationary (core) and migratory (rim) cells were harvested under an inverse microscope (Axiovert 100, Zeiss, NY) using a P20 pipette in four independent biological replicates. Forty individual dispersion assays (four 10-well slides) were collected and immediately isolated for RNA according to manufacturer's protocol (Mirvana Total RNA Kit, Ambion, Austin, TX). Core and rim cells were separately lysed with Lysis Binding Buffer. Total RNA quantity was assessed using a NanoDrop 2000c (Thermo Scientific, Wilmington, DE), and quality was assessed by Bioanalyzer RNA 6000 Nano LabChip Kit (Agilent Technologies, Palo Alto, CA).

### Microarray Data Collection and Analysis

All microarray data are compliant with the MIAME standards and are deposited in the GEO database. The accession numbers are GSE17708for the A549 cell line data; GSE21654 for the cell line panel; and GSE21566 for the migrating cell model. The induced-EMT and the cell line panel models were analyzed on the Affymetrix Human Genome U133 Plus 2.0 chip at the University of Michigan and the Van Andel Institute, respectively.

The resulting gene expression data from all model systems were analyzed using Microsoft Excel and Xenobase (26). A list of 587 genes of interest (see Table S1) was assembled using information from the Consortium for Functional Glycomics in addition to project-specific information. These genes include glycan-transferases (199 genes), glycoproteins (101 genes), lectins (112 genes), glycosidases (62 genes), nucleo-sugar transport (35 genes), nucleo-sugar synthesis (23 genes), golgi transport (8 genes), TGF pathway (19 genes), notch pathway (18 genes), and EMT markers (10 genes). 96.7% of these genes are represented on the Affymetrix Human Genome U133 Plus 2.0, and 92.2% are represented on the Agilent Whole Human Genome Microarray Kit, 4×44K. A Chi-square test was used to determine whether gene expression changes within this list occurred more frequently than all other changes.

For the analysis of TGFβ–induced EMT of PANC-1, a paired, two-tailed, t-test was performed between the triplicate experiments of each condition, with a p value less then 0.05 considered significant. Probes with mean absolute signal levels below 100 for both conditions were excluded. Analysis of TGFβ treated A549 used the pairwise ratios in transcript levels between the 0 hour and 72 hour time points. For the analysis of the 22 cell line panel, an unpaired, two-tailed, t-test was applied with a p value less than 0.01 considered significant. For the analysis of migrating Panc-1 and migrating MiaPaCa-2 cell lines, probes were excluded that showed less than a ±25% change in expression from rim to core, and genes were excluded that had multiple probes respectively showing both positive and negative changes. The results from the remaining probes were averaged for each gene. The data discussed in this publication have been deposited in NCBI's Gene Expression Omnibus and are accessible through GEO series expression numbers: GSE17708 & GSE23952 (A549 & Panc-1 TGFβ treatment assay, respectively), GSE21654 (22 Pancreatic Cancer Cell Line Panel), GSE21566 (Radial Migration Assay).

### Gene Expression Validation by RT-PCR

Synthesis of the first strand cDNA template closely followed the outlined protocol for the use of SuperScript II Reverse Transcriptase (Invitrogen, Carlsbad, CA). cDNAs were synthesized in a 20 µl reaction at RT. The first step used 5 µg RNA, random hexamer , dNTP mix, and ddH_2_O to a final volume of 12.5 µl incubated at 65°C for 5 minutes then quick chilled on ice. Once chilled, 5× first strand buffer stock, RNase inhibitor, DTT, and SuperScript II RT were added and incubated at RT for 10 minutes. After this initial incubation the reaction was carried out at 42°C for 1 hour and subsequently inactivated at 70°C for 10 minutes.

Gene-specific primers corresponding to selected genes from the Affymetrix U133 Plus 2.0 chip were designed using MacVector (MacVector Inc., Cary, NC) and synthesized (Integrated DNA Technologies, Inc., Coralville, IA). The following sequences were used. **GALNT3:** EX-S: 5′-ACAGTGTGCTCTATTCTTCACCTGC-3′. EX-AS: 5′-TTACGACAGCCGTGTAGTTCTCAG-3′. **ST3GAL2:** EX-S: 5′-CCTGCTGGTGTTCATCATGTCC-3′. EX-AS: 5′-GCACCTCATTGGTGTTGTGTGAC-3′. **ST3GAL6:** EX-S: 5′-CAGCTTTTGCCTCTCTGCTGAG-3′. EX-AS: 5′-TCTCCCAACTTCTTCTTCATGTCC-3′. **PMM1:** EX-S: 5′-CAAGCAGACCATCCAGAACCAC-3′. EX-AS: 5′-AGAGAACCTCAGCCCTTTGCCAG-3′. **MAN2B2:** EX-S: 5′-CCTAAACAGCCAGCAGGTCATC-3′. EX-AS: 5′-TGTCGTTCAGCGTGAGGTTGTAG-3′. **GMPPA:** EX-S: 5′-TCCTTGGCACTACGGCTAACAG-3′. EX-AS: 5′-GGGCTGAAAACACATCCTGCTC-3′. **ZEB1:** EX-S: 5′-AATCCCACCAAGTGCCAACC-3′. EX-AS: 5′-CATTCCATTTTCTGTCTTCCGC-3′. The Tm of these primers ranged from 54.5 to 61.5°C as reported by the manufacturer.

Each 50 µl reaction tube contained 0.5 µg cDNA template, 4µM primers, and the Qiagen reagents 1× PCR buffer, 1× Q solution, 0.2 mM dNTPs, and HotStar Taq DNA polymerase. The amplification program consisted of one activation round of 95°C for 15 minutes followed by 25 cycles of 94°C with a 50 second hold, 55°C with a 1 minute hold, and 72°C with a 50 second hold. The reaction was allowed to continue at 72°C for 10 minutes and held at 4°C. The PCR products were analyzed using a 1.5% agarose gel in 1× Tris base, acetic acid and ethylenediaminetetraacetic acid (EDTA) buffer (TAE). The gel bands were imaged and analyzed using the software programs Chemidoc and Quantity One (Bio-Rad, Hercules, CA).

### Western Blots

Cells were lysed in Radio-Immunoprecipitation Assay (RIPA) buffer containing protease inhibitor (Roche). The solution was centrifuged to remove the pellet. The protein-containing supernatant was then quantified using the microBCA assay (Thermo Scientific) and adjusted to equivalent concentrations prior to loading on 4–12% polyacrylamide gels (Criterion, BIO-RAD). The proteins from the gel were electrophoretically transferred to Polyvinylidene Fluoride (PVDF) membrane, which was blocked in 5% milk and incubated with primary antibody overnight at 4°C. After washing with Tris-Buffered Saline containing 0.5% Tween 20 thrice for 10 minutes each, the membrance was incubated with horseradish peroxidase (HRP)-conjugated secondary antibody for 1 hour and washed again. The membrane was incubated with substrate (SuperSignal West Pico, Thermo Scientific) for 5 minutes and visualized using BIO-RAD ChemiDOC XRS. The primary antibodies targeted E-cadherin (R&D Systems, mAb 1838), Vimentin (R&D Systems, AF2105), MRC2 (Abcam ab70132), and Actin (1–19) (Santa Cruz, SC-1616). The GALNT3 antibody was produced and generously donated by the laboratory of Dr. Henrik Clausen [51], [69].

### Sulfated Glycosaminoglycan Assay

The Alcian Blue Assay closely followed a previously established protocol [70] with minor alterations. Cells were lysed in Radio-Immunoprecipitation Assay (RIPA) buffer containing protease inhibitor (Roche). The solution was centrifuged to remove the pellet. The protein-containing supernatant was then quantified using the microBCA assay (Thermo Scientific) and adjusted to equivalent concentrations prior to analysis. All of the following reagents were purchased from Sigma, Samples were initially denatured in 3.3 M urea, 0.1% H_2_SO_4_, and 0.25% octylphenoxypolyethoxyethanol (Triton X-114) for 30 minutes at room temperature. Alcian Blue stain solution (0.07% Alcian Blue/0.1% H_2_SO_4_/0.25% Triton X-114) was then added to give final concentrations of 0.05% Alcian Blue, 0.56 M urea, 0.1% H_2_SO_4_, and 0.25% Triton X-114. Samples were then left at 4°C overnight to allow precipitation of Alcian Blue bound species. The concentration of H_2_SO_4_ used should give a pH between 1.5 and 2.0 to allow for the majority of species carrying negative charge to be sulfate residues for specific binding by the cationic dye. The next day the samples were centrifuged at 12,000×g for 15 minutes and the supernatant containing unbound dye was discarded. The pellets containing the dye bound molecules were re-suspended in 1 mL of a solution of 40% dimethylsulfoxide (DMSO) and 0.07M MgCl_2_ then centrifuged at 12,000×g for 15 minutes and the supernatant was once again discarded. The remaining pellet was re-dissolved in 500 µL of a solution of 5M urea, 33% 1-propanol, and 0.25% Triton X-114 to measure absorbance. Measurements for absorbance at 600 nm were performed in triplicate using a BioPhotometer (Eppendorf). A solution of 5M urea, 33% 1-propanol, and 0.25% Triton X-114 was used as a blank. Heparin (H3393, Sigma) was used as a positive control. The statistical analyses and graphing were performed in Microsoft Excel.

## Supporting Information

Table S1 A list of 587 genes of interest was assembled using information from the Consortium for Functional Glycomics in addition to project-specific information. These genes include glycan-transferases (199 genes), glycoproteins (101 genes), lectins (112 genes), glycosidases (62 genes), nucleo-sugar transport (35 genes), nucleo-sugar synthesis (23 genes), golgi transport (8 genes), TGF pathway (19 genes), notch pathway (18 genes), and EMT markers (10 genes).
(0.06 MB XLSX)

Figure S1 TGFβ-induced EMT in PANC-1. PANC1 cells were cultured in serum-free media for 24 hours followed by treatment with (A) control media or (B) 5 nM TGFβ. The photomicrographs were taken after 72 hours at 10× magnification.
(1.13 MB TIF)

Figure S2 Expression profiles of EMT-associated genes in the panel of pancreatic cancer cell lines. The cells lines were grouped according to morphological characteristics. The expression level of each gene was log transformed (base 10) and median centered by row. The value of each square corresponds to the color bar at top. The levels of the ZEB1 gene most clearly associated with morphology.
(0.47 MB TIF)
