# Supplementary figures and images for: Glycogene Expression Alterations Associated with Pancreatic Cancer Epithelial-Mesenchymal Transition in Complementary Model Systems
Source: PLoS One. 2010 Sep 27;5(9):e13002. doi: 10.1371/journal.pone.0013002 (PMC2946336; doi:10.1371/journal.pone.0013002)

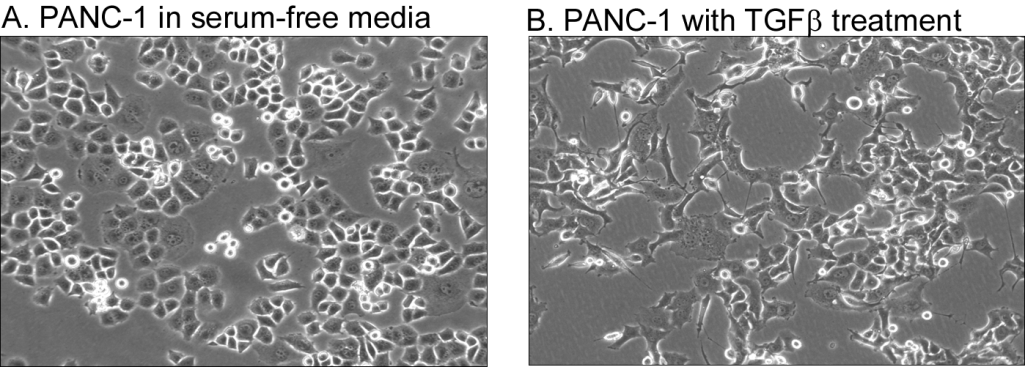

Supplement: Figure S1 — TGFβ-induced EMT in PANC-1. PANC1 cells were cultured in serum-free media for 24 hours followed by treatment with (A) control media or (B) 5 nM TGFβ. The photomicrographs were taken after 72 hours at 10× magnification. (1.13 MB TIF) [file pone.0013002.s002.tif]

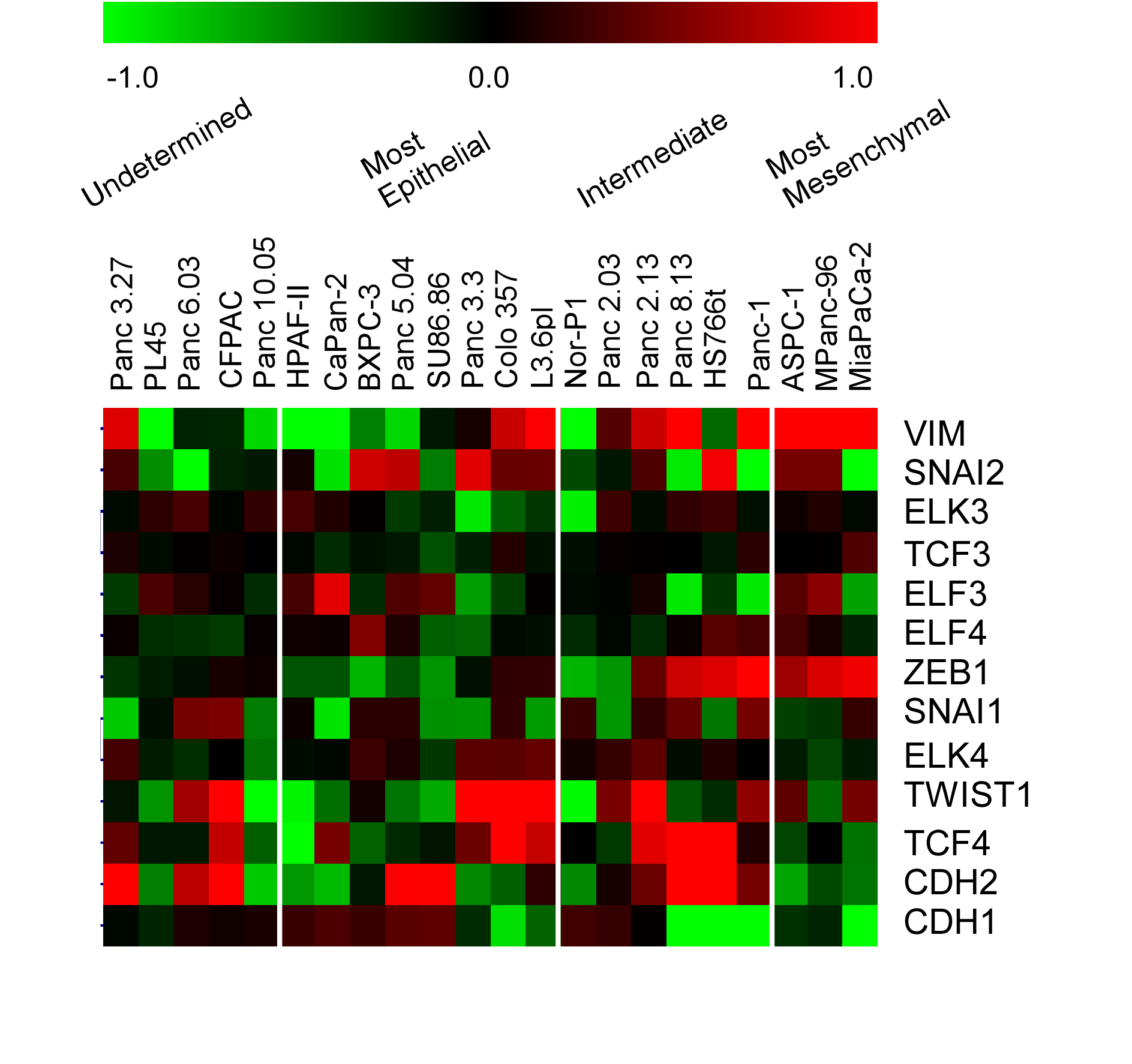

Supplement: Figure S2 — Expression profiles of EMT-associated genes in the panel of pancreatic cancer cell lines. The cells lines were grouped according to morphological characteristics. The expression level of each gene was log transformed (base 10) and median centered by row. The value of each square corresponds to the color bar at top. The levels of the ZEB1 gene most clearly associated with morphology. (0.47 MB TIF) [file pone.0013002.s003.tif]
